# The Artesian Well at Naples

**Published:** 1844-12-14

**Authors:** 


### THE ARTESIAN WELL AT NAPLES.

The boring has arrived at the depth of 108 yards. It has traversed sixty yards of yellow tufa; next it has pierced a gray tufa of much older date, and the marine formations and sands intermixed with thiu layers of pumice, are beginning to appear. The temperature augments very slowly.—*Med. Times.*

